# Chemically crosslinked hyaluronic acid-chitosan hydrogel for application on cartilage regeneration

**DOI:** 10.3389/fbioe.2022.1058355

**Published:** 2022-12-19

**Authors:** Sandra Escalante, Gustavo Rico, José Becerra, Julio San Román, Blanca Vázquez-Lasa, Maria Rosa Aguilar, Iván Durán, Luis García-Fernández

**Affiliations:** ^1^ Department of Cell Biology, Genetics and Physiology, Faculty of Science, University of Malaga, Malaga, Spain; ^2^ Centro de Investigación Biomédica en Red de Bioingeniería, Biomateriales y Nanomedicina (CIBER-BBN), Instituto de Salud Carlos III, Madrid, Spain; ^3^ Grupo de Biomateriales, Departamento de Nanomateriales Poliméricos y Biomateriales, Instituto de Ciencia y Tecnología de Polímeros (ICTP), CSIC, Madrid, Spain

**Keywords:** hydrogel, cartilage regeneration, microfracture, hyaluronic acid, chitosan, chondroitin sulfate, diisocyanate

## Abstract

Articular cartilage is an avascular tissue that lines the ends of bones in diarthrodial joints, serves as support, acts as a shock absorber, and facilitates joint’s motion. It is formed by chondrocytes immersed in a dense extracellular matrix (principally composed of aggrecan linked to hyaluronic acid long chains). Damage to this tissue is usually associated with traumatic injuries or age-associated processes that often lead to discomfort, pain and disability in our aging society. Currently, there are few surgical alternatives to treat cartilage damage: the most commonly used is the microfracture procedure, but others include limited grafting or alternative chondrocyte implantation techniques, however, none of them completely restore a fully functional cartilage. Here we present the development of hydrogels based on hyaluronic acid and chitosan loaded with chondroitin sulfate by a new strategy of synthesis using biodegradable di-isocyanates to obtain an interpenetrated network of chitosan and hyaluronic acid for cartilage repair. These scaffolds act as delivery systems for the chondroitin sulfate and present mucoadhesive properties, which stabilizes the clot of microfracture procedures and promotes superficial chondrocyte differentiation favoring a true articular cellular colonization of the cartilage. This double feature potentially improves the microfracture technique and it will allow the development of next-generation therapies against articular cartilage damage.

## 1 Introduction

Articular cartilage (AC) is an avascular connective tissue located in the diarthrodial joints covering the surface of bones, their main function is to provide a low friction surface for transmitting the mechanical load between bones (Adams et al., 2009).

AC is formed by chondrocytes distributed within an extracellular matrix (ECM). This ECM is composed of collagen, mainly of type II, glycosaminoglycans (GAGs) and proteoglycans (Mow et al., 1992). The main characteristics of this tissue, in addition to its peculiar structure and topography (Becerra et al., 2010), are its low metabolism and the absence of blood vessels, nerves and lymphatic systems. These features could be the reasons for its limited capability of repair and regeneration, especially for large defects (>3 mm^2^) (Doherty, 1992; Armiento et al., 2018).

Cartilage damage, one of the major skeletal morbidities in our society, is usually produced by repeating loads, sudden impact, foreign bodies or damage in other connective tissue, but also because of the natural degeneration due to the decrease of biological and biomechanical properties through aging. In both cases, the progressive degeneration of AC is the most common form of joint disease and represents one of the main causes of osteoarthritis (OA) (Lawrence et al., 2008).

Currently there are several options for the treatment of cartilage defects, but most of the current treatments are focused on symptom relief (Mollon et al., 2013):

• Non-surgical treatment: Oral analgesia, physiotherapy, intraarticular injection of GAGs, platelet rich plasma (PRP) and/or anti-inflammatory drugs (Pontes-Quero et al., 2019; García-Fernández et al., 2020).
• Arthroscopic chondroplasty: The damaged cartilage, loose bodies, and chondral fragments are removed (mechanical saver devices, radiofrequency ablation…) to produce a smooth chondral surface and decrease the mechanical joint irritation (Liptak and Theodoulou, 2017).
• Pridie Drilling or Microfracture (MF): Minimally invasive procedure that drills the subchondral bone at the defected site with the objective of releasing osteoprogenitor cells into the defect (Dehghani and Fathi, 2017).
• Autologous or allogenic osteochondral grafting: This technique uses allogenic or autologous osteochondral tissue to fill the defect (Dehghani and Fathi, 2017). Autologous Chrondrocytes Implant (ACI): Implantation of chondrocytes coming from an enzymatically treated biopsy from patient cartilage (Mistry et al., 2017).
• Matrix Autologous Chondrocytes Implant (MACI): Similar to ACI, but the cells are growing in a matrix, and this matrix is implanted to the patient (Mistry et al., 2017).

A typical lesion of professional athletes and sport players exposed to great efforts consists on articular damage with an average size of around 2–3 mm^2^ without affection of the subchondral bone. In this situation, the most used procedure is the microfracture (Sledge, 2001). Microfracture is an inexpensive technique, it presents a quick recovery time and reports positive result in pain relief and joint functionality (Mollon et al., 2013). This technique consists of the micro-perforation of the subchondral bone, allowing bleeding. The blood coming from the subchondral bone fills the damage and forms a clot with high levels of stem cells and growth factors (Frehner and Benthien, 2018). This clot acts as a scaffold for the development of the new cartilage. However, this technique often results in the formation of fibrocartilage with lower biomechanical properties than those of hyaline cartilage. Other problems with this procedure are the instability of the clot (resulting in a deficient integration with the surrounding tissue) and the fast rate of degradation (not allowing the complete regeneration of the cartilage) (Chung and Burdick, 2008).

Recent advances in cartilage repair focus on the use of scaffolds enhancing the biochemical and biomechanical properties of the cartilage regeneration process. In these studies, different biomaterials have been proposed as scaffolds for cartilage regeneration like gellan gum, polyglucosamine/glucosamine carbonate hydrogel or fibrin-hyaluronan matrix among others (Nehrer et al., 2008; Oliveira, 2010; Vilela et al., 2018; Pipino et al., 2019).

In this article, we present the development of an adhesive polymer scaffold based on hyaluronic acid (HA) and chitosan (Ch) to fit into a cartilage defect after a microfracture procedure with the capacity to stabilize the blood clot in the cartilage. HA is non-immunogenic natural polymer with a unique viscoelastic nature and one of the main components of the hyaline cartilage. (Chartrain et al., 2022). It is commonly used in intra-articular injections to stimulate and repair damaged cartilage because it promotes cell adhesion and proliferation and presents anti-inflammatory properties as well as non-immunogenicity (Shiroud Heidari et al., 2023). However, hydrogels made of HA present quick degradation rates, for this reason it is necessary to modify them to enhance their degradation properties. Ch is a cationic polysaccharide easy to obtain from crustacean shells. It is widely used as biomaterial due to its low toxicity and good biocompatibility and degradability (Shigemasa and Minami, 1996). Ch also present good adhesive properties to tissue and hemostasis, these properties allow a normal clot formation and impeding clot retraction (Shive et al., 2006; Chen, 2018; Pereira et al., 2018). Different studies demonstrate the efficacy of the marrow-derived mesenchymal stem cells (MSC) to migrate into the defect, proliferate, fill cavities and differentiate creating new cartilage (Shapiro et al., 1993; Anraku, 2009). Chondroitin sulfate (CS) is one of the principal GAGs presents in the cartilage ECM. CS present anti-inflammatory activity promotes cell differentiation and protect cartilage from ECM degradation due to regulation of the metabolism of cartilage tissue (Lafuente-Merchan, 2022). This together with the fact that blood drained from subchondral bone contains MSC constitutes the hypothesis to study differentiation processes in our novel biomaterial, testing it on this stem cell population. Our results demonstrate that our biomaterial composed of HA, Ch and Chondroitin Sulfate (HAChCS) not only favors MSC settlement but also induces their differentiation into cartilage tissue highly resembling the articular cartilage in its surface.

## 2 Materials and methods

### 2.1 Materials and reagents

High molecular weight (800–100 kDa) sodium hyaluronate (HA, pharmaceutical grade) and sodium chondroitin sulfate from bovine (injectable grade) were kindly supplied by Bioiberica (Barcelona, Spain). Medical grade Ch of low molecular weight (260 kDa) and 90.5% of deacetylation degree was purchased from Altakitin (Lisbon, Portugal). L-lysine diisocyanate (97%) was purchased from BOC Sciences (NY, United States), acetic acid and Pluronic F-127 was purchased from Sigma-Aldrich (St. Louise, United States).

All products were used as received without further purification.

### 2.2 Preparation of hydrogel scaffolds

Hyaluronic acid-Chitosan scaffolds (HACh) were prepared by crosslinking with L-lysine diisocyanate (LDI). HA was dissolved in an aqueous solution of Pluronic F-127 (1% w/v) and acetic acid (1% v/v) at a concentration of 20 mg/ml. Pluronic F-127 was added as a surfactant to preserve the porosity until the hydrogel is formed. Ch was added in a 1:1 relationship and stirred at 37°C until complete dissolution. In the case of hydrogels containing chondroitin sulfate 4 mg/ml of CS were added at this point. The final solution was mixed with LDI in a NH_2_/LDI relationship of 1:5, dispersed with an UltraTurrax at 15,000 rpm for a minute and placed on a Teflon mold. The dispersion was allowed to crosslink at 37°C in a humidified chamber for 1 h. The final scaffold was washed with distilled water and freeze-dried to obtain the final scaffold.

### 2.3 Physicochemical characterization

#### 2.3.1 Scanning electron microscopy and energy dispersive spectroscopy analysis

The morphology of the scaffolds was analyzed by analytic scanning electron microscopy (SEM) using a JEOL JSM-6010LV (Tokyo, Japan). Prior analysis all samples were sputter-coated with gold using a Leica EM ACE600 coater (Leica Microsystems, Wien, Austria). Elemental composition was performed by energy dispersive spectroscopy (EDS, Bruker XFlash model with detector 5030) installed in the SEM. Three independent areas were selected in the scaffolds.

#### 2.3.2 Fourier transform infrared attenuated total reflectance (ATR-FTIR)

ATR-FTIR spectroscopy was performed in a Perkin Elmer Spectrum One FTIR spectrometer using 32 scans, and a resolution of 4 cm^−1^.

#### 2.3.3 Dynamic mechanical analysis

Rheology measurements were carried out in a stress-controlled oscillatory rheometer ARG2 (TA Instruments) using parallel plate geometry, using a 20 mm diameter steel plate. Oscillatory frequency sweeping test of scaffolds was conducted with a frequency scanning from 0.01 to 20 Hz at 0.1% strain and 25°C. Five replicates of each sample swollen in phosphate buffer were evaluated.

### 2.4 Swelling-degradation profile and chondroitin sulfate release

The gravimetric swelling ratio is defined as the fractional increase in the weight of the hydrogel due to water absorption. The gravimetric swelling capacity was measured in rounded scaffolds (*d* = 11 mm). The dried scaffolds were weighed ( $m_{0}$ ) and then incubated in PBS at 37°C for 1 day. At regular intervals, the scaffolds were removed from the PBS solution, the excess of water was eliminated with filter paper and the samples were weighed ( $m_{t}$ ). The gravimetrically swelling percentage was calculated as:

$$Gravimetric\;Swelling\;\left(\%\right)=100\times \left(m_{t}-m_{0}\right)/m_{0}$$ (1)

The volumetric swelling ratio is defined as the volume increase of the hydrogel due to water absorption. The volumetric swelling capacity was measured in rounded scaffolds (*d* = 11 mm). The dimensions were measured ( $V_{0}$ ) prior to incubation in PBS at 37°C for 1 day. At regular intervals, the scaffolds were removed from the PBS solution and the samples were measured ( $V_{t}$ ). The volumetric swelling percentage was calculated as:

$$Volumetric\;Swelling\;\left(\%\right)=100\times \left(V_{t}-V_{0}\right)/V_{0}$$ (2)

The stability of HACh and HAChCS scaffolds was carried out by immersing the membranes in 5 ml of PBS solutions and incubating at 37°C. After different periods of time the samples were removed from de PBS, washed with distilled water to eliminate the remaining salts and freeze-dried to obtain the weight of the sample at time t. The weight loss of the sample can be determined as:

$$Weight\;loss\;\left(\%\right)=100\times \left(m_{i}-m_{t}\right)/m_{i}$$ (3)

where $m_{i}$ is the initial weight of the sample and $m_{t}$ is the weight of the degraded sample at each time point.

For release experiments, scaffolds containing CS were incubated in PBS at 37°C. At regular intervals, the supernatant liquid was collected and analyzed by UV (Nanodrop One, Thermofisher). The absorbance value at 222 nm where compare with a calibration curve previously calculated to obtain the concentration of CS on the supernatant liquid.

### 2.5 Adhesion strength test

The bioadhesion properties of the scaffolds to bone tissue were tested using a modification of the ASTM F2258-05 method. Chicken keel bone were cut into homogeneous samples of 12 mm × 12 mm × 3 mm (length, width, thickness). To simulate the microfracture procedure five holes (*d* = 0.1 mm) were made on the surface of the bone. The corresponding scaffold were swollen in different conditions: PBS, fibrin glue (FG) or with chicken blood, and put in contact for 1 min with the bone. This time allow the coagulation of the fibrin glue or the formation of the clot. Adhesion strength was tested in a Universal Testing Machine (UTM, Instron model 3366) comparing drilled and non-drilled bones. The data were collected using a 100 N load cell and a loading rate of 5 mm/min.

### 2.6 *In vitro* toxicity and cell adhesion experiments

#### 2.6.1 Cell cultures

Primary human osteoblast from femoral bone tissue (hOBs), and human articular chondrocytes from knee articular cartilage (hACs) (Innoprot, Derio, Spain) were used in this study. hOBs were grown in DMEM/F12 medium (Dulbecco’s modified Eagle’s medium/F12; Gibco^®^, Life Technologies, Carlsbad, United States) supplemented with 10% (v/v) fetal bovine serum (FBS; Life Technologies, Carlsbad, CA, United States) and 1% (v/v) penicillin/streptomycin solution (final concentration of penicillin 100 units/ml and streptomycin 100 mg/ml, Life Technologies, Carlsbad, CA, United States). Both cell lines were cultured until 90% of confluence at 37°C and 5% CO_2_, changing the culture medium every 2–3 days to be ready for the different assays.

#### 2.6.2 Cytotoxicity assay

An MTT test ((3-(4,5-dimethylthiazol-2-yl)-2,5-diphenyltetrazolium bromide)) was used to indirectly analyze the cytotoxicity of the different scaffolds. Rounded scaffold (*d* = 11 mm) were immersed in 5 ml of FBS-free culture medium and incubated at 37°C. Thermanox^®^ (TMX) discs (Nunc^®^, Thermofisher) were used as negative control. Aliquots of the supernatant medium were taken at 1, 2, 7, 14 and 21 days under sterile conditions and replaced with fresh medium. The samples were kept frozen until use. hObs and hACs were seeded separately in a 96-well plate at 9 × 10^4^ cells/ml of density and incubated for 24 h in complete medium. Then, the medium was substituted by the extracts and incubated at 37°C. After 24 h the medium was replaced by a solution of MTT (0.5 mg/ml) in warm FBS-free medium and the cells were incubated at 37°C for 3–4 h. Mediua containing MTT were removed and DMSO was added to dissolve the formazan crystals formed in the cells. The absorbance of the medium was measured with a Biotek Synergy HT detector at 570 nm and a reference wavelength of 630 nm. Cell viability was calculated as:

$$Cell\;Viability\;\left(\%\right)=100\times \left(OD_{S}-OD_{B}\right)/\left(OD_{C}-OD_{B}\right)$$ (4)

where $OD_{S}$ , $OD_{B}$ and $OD_{C}$ are the optical density (OD) of formazan production for the sample, blank and control, respectively.

#### 2.6.3 Cell adhesion and proliferation assay

Cell adhesion and proliferation on the scaffolds were tested by the Alamar Blue assay following the manufacturer’s instructions. Cells were seeded at 4 × 10^5^ cell/ml on the previously swollen scaffolds and incubated at 37°C in a humidified atmosphere with 5% CO_2_. Cell proliferation was measured at determinate times (1, 4, 7, 14 and 21 days) by replacing the culture medium with a 10% solution of AB in phenol red free DMEM medium. After 4 h the solution was transferred into a 96-well plate and the fluorescence was measured in a microplate reader (Synergy HT, BioTek, Instruments, United States). The excitation wavelength was 530 nm and the emission was recorded at 590 nm.

### 2.7 *In vitro* evaluation of cartilage formation on HACh scaffolds

#### 2.7.1 Cell culture

C3H10T1/2 cell line (mouse: ATTC, CCL-226) was cultured in 75-cm^2^ polystyrene culture flask in Dulbecco’s Modified Eagle’s Medium—low glucose (Sigma, D5546-1X500ML) supplemented with 10% fetal bovine serum (GIBSO, F7524-500ml), 1% Penicillin-Streptomycin (Sigma, P4333-100ML) and 1% Glutamax (GIBCO, 35050-038) in a humidified incubator at 37°C with 5% CO_2_. Passages were performed at 80% confluence with Trypsin-EDTA 0.05% (Thermofihser, 25300096) and cells were always used in the 15th passage.

#### 2.7.2 Scaffold seeding

Ch , HACh and HAChCS disks were rinsed in 70% ethanol for 20 min in agitation and punched into cylinders of 3 mm diameter. The pieces were sterilized in 70% ethanol for 2 h, rinsed in PBS for 30 min three times and maintained in culture medium at 4°C until use.

For the scaffold seeding, the cylinders obtained from each material were left at room temperature for 1 h, partially dried with a cell strainer (DICSA) and placed on a non-treated culture petri dish. C3H10 cells were trypsinized, resuspended in culture media and 1 × 10^5^ cells were seeded in each 3 mm cylinders of biomaterial. The scaffolds were incubated at 37°C and 5% CO2 for 2 h to allow adhesion, adding 3 µl of culture media every 30 min. Then, the cylinders were placed in the center of a well in a standard 48-well plate (Jet BioFil) and incubated in culture media in a humidified incubator at 37°C with 5% CO_2_ for 1 and 2 weeks. Culture media were replaced every 2–3 days the first week and 3–4 days the second week. Micromass of C3H10 cells was also obtained as controls by centrifugation of 1 × 10^5^ cells at 1.800 rpm at 5 min, removing the supernatant and adding 250 µl of culture media. Micromass was kept under the same conditions as Ch scaffolds. To stimulate differentiation, the biomaterials were incubated in culture media containing 100 ng/ml BMP-2 (R&D Systems, 355-BM-050) at 37°C in a humidified incubator with 5% CO_2_ for 1 and 3 weeks. Culture media were replaced every 2–3 days the first week and 3–4 days the second week.

#### 2.7.3 Histology

After the differentiation assays, the scaffolds were rinsed with PBS and fixed with 4% paraformaldehyde at 4°C overnight. Then, they were washed in PBS, dehydrated with a graded series of ethanol, embedded in paraffin and sectioned at 15 μm thick. For histological analysis, sections were rehydrated and stained with Alcian Blue and Safranin-O.

#### 2.7.4 Immunohistochemistry

For immunohistochemistry, sections were treated with citrate buffer for antigen retrieval and quenched by peroxidase solution. After blocking with goat serum, Aggrecan antibody (13880 Proteintech) was incubated overnight 1:100. Sections were developed with histostain plus kit with DAB as a chromogen (Invitrogen). Negative controls for primary antibody were included. Briefly, control slides were exposed to antigen retrieval and maximum DAB development to confirm no unspecific signal development (Supplementary Figure S3).

#### 2.7.5 Real-time polymerase chain reaction (qPCR)

Total RNA from the cultured scaffolds was extracted using the Trizol reagent (ThermoFisher, 15596018) and RNA concentrations were determined using Nanodrop 2000 spectrophotometer (Thermo Fisher Scientific). Complementary DNA (cDNA) was amplified with the PrimeScript™ RT Master Mix (Takara, RR036A) and used for qPCR, which was performed in triplicates by using the TB Green^®^ Premix Ex Taq™ (Takara, RR420L) on the Bio-Rad C1000 Thermal Cycler (Bio-Rad). The housekeeping gene, β-actin, was used for gene expression normalization and the fold change expression was calculated using the 2^−ΔΔCT^ method. Primer sequences were purchased from Invitrogen and listed in Table 1.

**TABLE 1:** Sequences of qRT-PCR primers.

Gene	Forward	Reverse
β-actin	5′-GGA​GAT​TAC​TGC​CCT​GGC​TCC​TA-3′	5′-GAC​TCA​TCG​TAC​TCC​TGC​TTG​CTG-3′
Sox9	5′-GAG​GCC​ACG​GAA​CAG​ACT​CA-3′	5′-CAG​CGC​CTT​GAA​GAT​AGC​ATT-3′
Acan1	5′-GGT​CAC​TGT​TAC​CGC​CAC​TT-3′	5′-CCC​CTT​CGA​TAG​TCC​TGT​CA-3′

### 2.8 Statistical analysis

Results are given as mean and standard deviation (minimum n = 4). Data from the different groups were compared in pairs with ANOVA test. All the statistical analyses were performed using the Origin 9 (Origin Lab corporation, Northampton,United States). The significance level was set at *p < 0.05, **p < 0.01 and ***p<0.001.

## 3 Results and discussion

### 3.1 Scaffold synthesis and characterization

**FIGURE 1:**
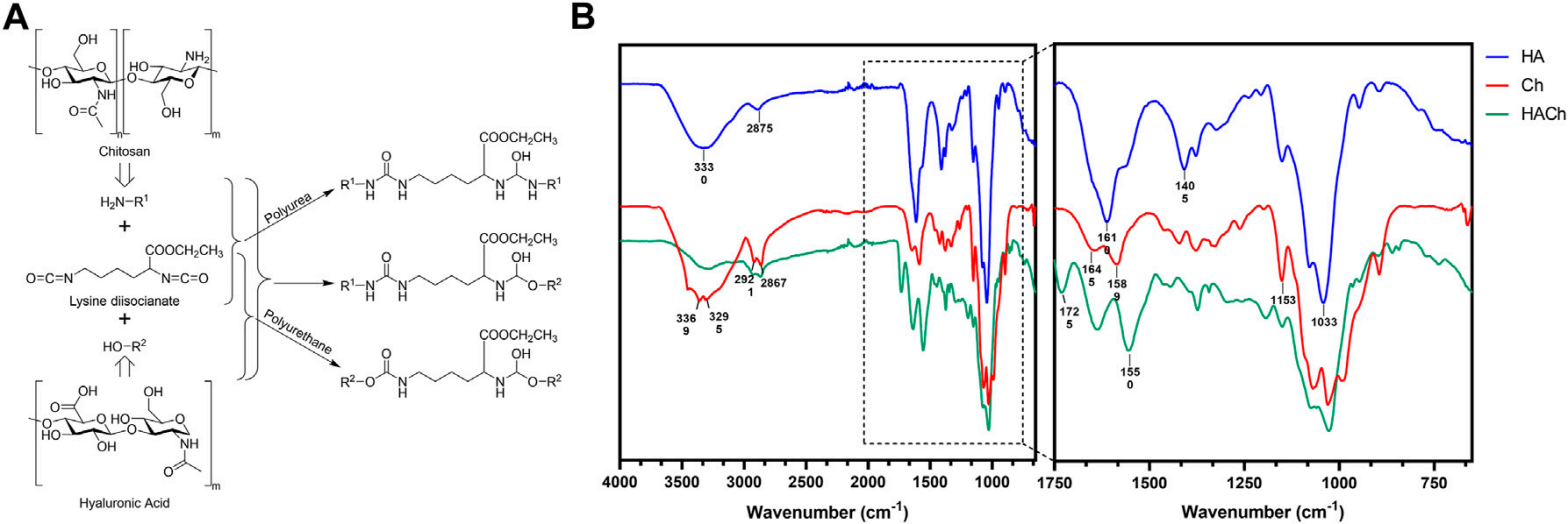
**(A)** Proposed scheme reaction for HACh scaffolds. **(B)** ATR-FTIR spectra of HA, Ch and HACh scaffolds.

The use of lysine diisocyanates as crosslinker allows different reactions between Ch and HA as is shown in Figure 1A.

The presence of the different reactions could be elucidated by ATR-FTIR spectra of HA, Ch and HACh scaffolds (Figure 1B). HA spectrum showed the most characteristics bands: the broad band around 3330 cm^−1^ is associated with the intra- and intermolecular stretching vibration of –OH group. The signal at 2,875 cm^−1^ correspond to the stretching of –CH_2_ group; the bands from 1,610 and 1,405 cm^−1^ are correlated with symmetric and asymmetric vibration of COO^−^ group. Finally, the peak from 1,033 cm^−1^ is related with the C–O–C from the saccharide units (Vasi, 2014).

Ch spectrum showed also their typical bands: The strong bands at 3369 and 3295 cm^−1^ correspond to N-H and O-H stretching. The bands at 2,921 and 2,867 cm^−1^ correspond to C-H stretching (symmetric and asymmetric). At 1,654 cm^−1^ correspond to C=O stretching of amide I and at 1,589 cm^−1^ correspond to the N-H bending of the primary amine. The absorption band at 1,153 cm^−1^ can be attributed to asymmetric stretching of the C-O-C bridge.

Analyzing the HACh spectrum, the signal of HA at 1,610 and 1,405 cm^−1^ (stretching of carboxylate group) was vanished, indicating that the conjugate was formed through the COO^−1^ group. In the case of the Ch, the signal corresponding to the primary amine (1,589 cm^−1^) also disappears in the HACh spectrum, indicating the formation of the crosslinking between the amine and isocyanate group. In the HACh spectrum, new signals appear at 1,550 and 1725 cm^−1^, which are in the range of the secondary amine bands and C=O stretching in polyurethanes and polyureas. These results demonstrate the formation of a stable scaffold.

**FIGURE 2:**
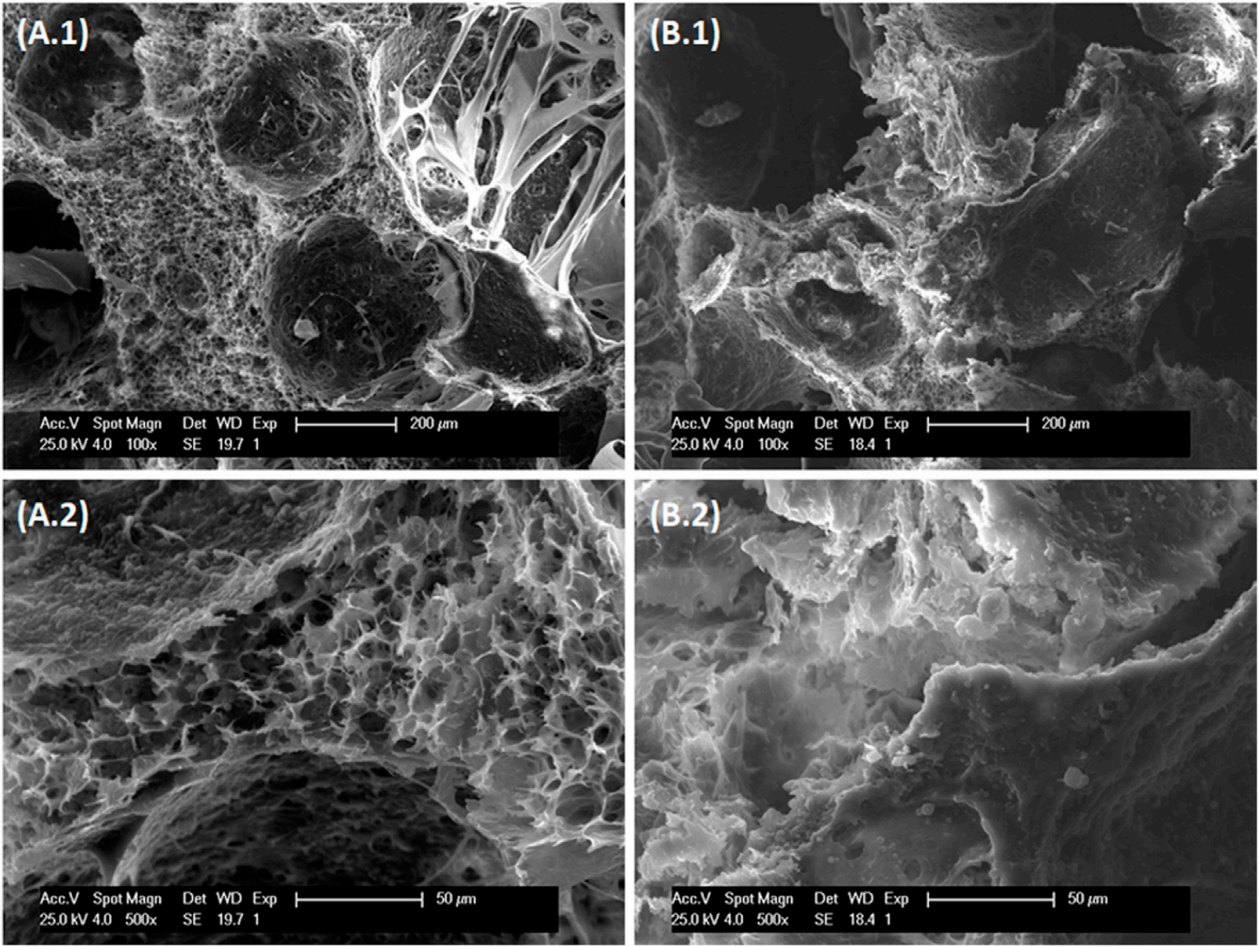
SEM images of **(A)** HACh and **(B)** HAChCS at different magnifications: **(A1,B1)** ×100 and **(A2,B2)** ×500.

The use of the UltraTurrax in the synthesis process produced scaffolds with a high porosity due to the introduction of microbubbles in the system (Figure 2).

In HACh scaffolds (Figures 2A1, A2), we can observe the presence of two different kinds of microstructures: a macroporosity (≈200 μm) and a microporosity (≈10 μm). The microporosity is partially occulted on CS loaded scaffolds (Figures 2B1, B2). In this case, CS is deposited on the microstructure partially covering it. The presence of CS on the surface was corroborated using EDS (Supplementary Table S1) that detected the presence of sulfur coming from the CS. This porosity pattern will allow the colonization of the scaffold by the cells contributing to the full regeneration of the damage.

Mechanical properties of HACh and HAChCS swollen scaffolds were analyzed by rheology (Supplementary Figure S1A). Storage and loss moduli exhibited a visco-elastic solid behavior close to a gel-like. HAChCS scaffolds showed lower storage moduli values in comparison with not loaded scaffolds.

Previous studies define the behavior of articular cartilage using the complex modulus (G*) and define different ranges: immature cartilage: G* < 1 MPa and mature cartilage 5 < G*< 16 MPa at low frequencies (Perni and Prokopovich, 2020). The values obtained for our scaffold (Supplementary Figure S1B) are in the range of an immature cartilage (0.5–0.8 MPa) (Perni and Prokopovich, 2020) being adequate for the regeneration of new tissue when it is applied in articular defects currently treated with microfracture procedure.

### 3.2 Swelling, degradation and CS release

**FIGURE 3:**
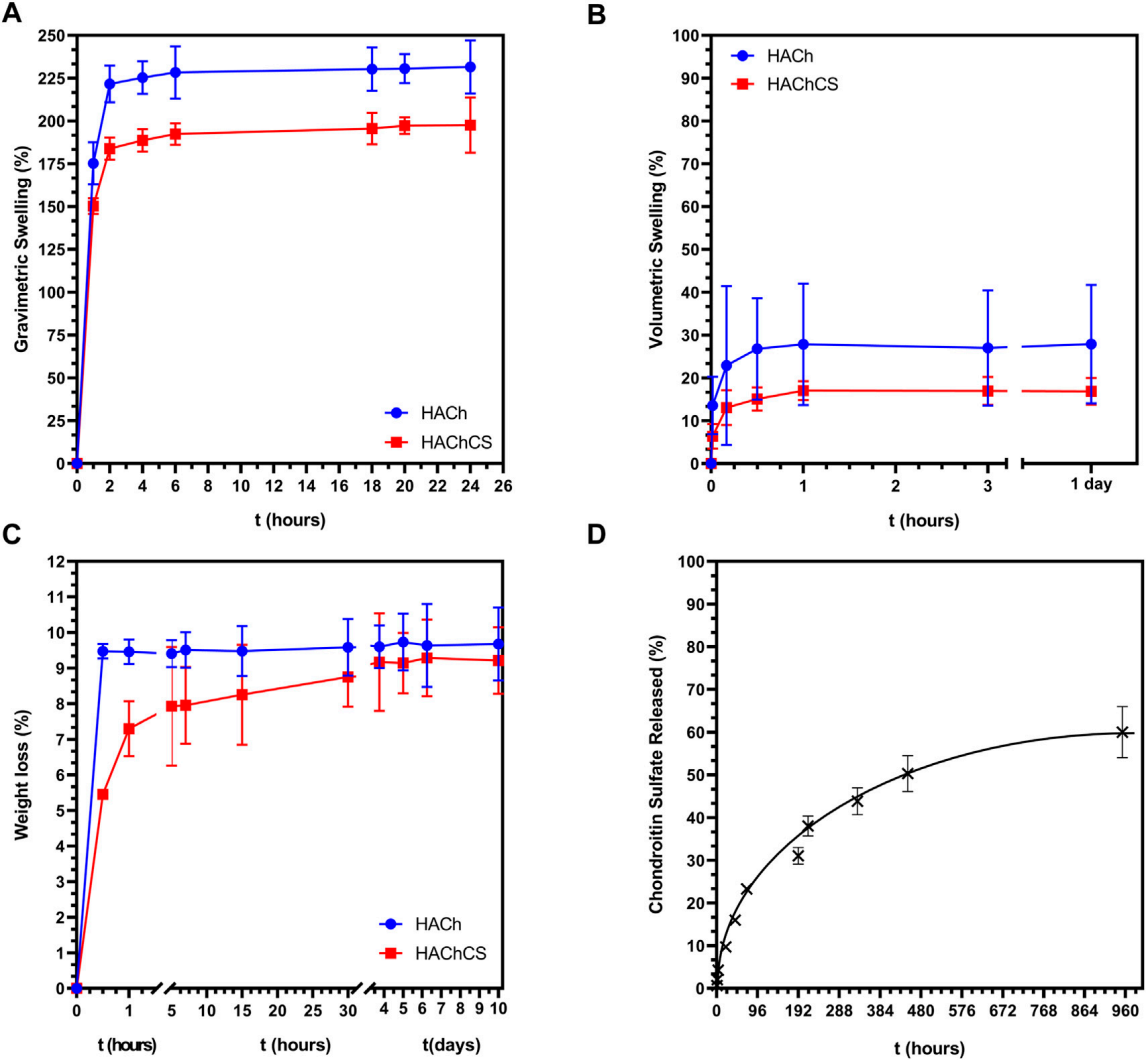
**(A,B)** Swelling behavior of HACh and HAChCS scaffolds in PBS at 37°C. **(C)** Degradation of HACh and HAChCS scaffolds in PBS at 37°C. **(D)** Chondroitin sulfate released from HAChCS scaffolds in PBS at 37°C.

Liquid adsorption capacity is an essential feature and determine the ability of the scaffold to interact with the blood coming from the microfracture process. This adsorption needs to be fast to stabilize the blood clot and adsorb the growth factors and stem cells coming from the blood. Figure 3A shows the swelling capacity of the HACh and HAChCS scaffolds. The maximum water-uptake equilibrium is reached after 4–6 h, reaching values of 225% for HACh and 200% for HAChCS. High initial swelling degrees are necessary for ensuring an appropriate source of nutrients to the whole scaffold, but too high swelling degrees can compromise scaffold integrity (Mora-Boza, 2020).

Volumetric swelling is an important scaffold characteristic to determine the ability of the gel to remain inside the microfracture holes. High volume changes could affect the stability of the gels once they are implanted in the defect. In our case (Figure 3B), the volume increase values are only 27% for HACh scaffolds and 17% for HAChCS scaffolds, being the maximum variation in the diameter of the scaffold of 0.55 ± 0.08 mm which ensures the permanence of our hydrogels in the defect.

Figure 3C shows the degradation profile of the scaffolds submerged in PBS at 37°C. Both scaffolds showed an initial weight loss due to the release of not integrated polymer chains and then they were stable for more than 2 months. The HAChCS presented a more sustained degradation may due to polyelectrolyte interactions between chondroitin sulfate and Ch (Rodrigues et al., 2016). Figure 3D shows the release of chondroitin sulfate from the HAChCS scaffold. The release profile shows an initial fast release of CS (10%) that was progressively increasing with the time until reaching a value of 40% after 40 days. This behavior is typical for systems that are capable of swelling and whose release profile fit the Korsmeyer-Peppas model Eq. 5:

$$\frac{M_{t}}{M_{\infty }}=k\cdot t^{n}$$ (5)

were $\frac{M_{t}}{M_{\infty }}$ is the fraction of CS released at time t, k is the release rate constant and n is the diffusional exponent which is indicative of the transport mechanism (Ritger and Peppas, 1987). Assuming our scaffold as a thin film we fit our data to Eq. 5 obtaining a value of *n* = 0.4982. Table 2 shows model values for *n* of the Peppas equation and that of the HAChCS sample.

**TABLE 2:** Experimental and theoretical exponent n of the Peppas equation for drug release mechanism.

HAChCS	Thin film	Cylinder	Sphere	Drug release mechanism
Exponent, *n*				
0.4982	0.5	0.45	0.43	Fickian diffusion
	0.5 < *n* < 1.0	0.45 < *n* < 0.85	0.43 < *n* < 1.45	Anomalous transport
	1.0	0.89	0.85	Case-II transport

Then, our system fit to thin film were the CS is released by Fickian diffusion from the swollen matrix. The CS that is homogeneously distributed in the polymer matrix is released by diffusion through the pores of the matrix.

### 3.3 Adhesion strength test

**FIGURE 4:**
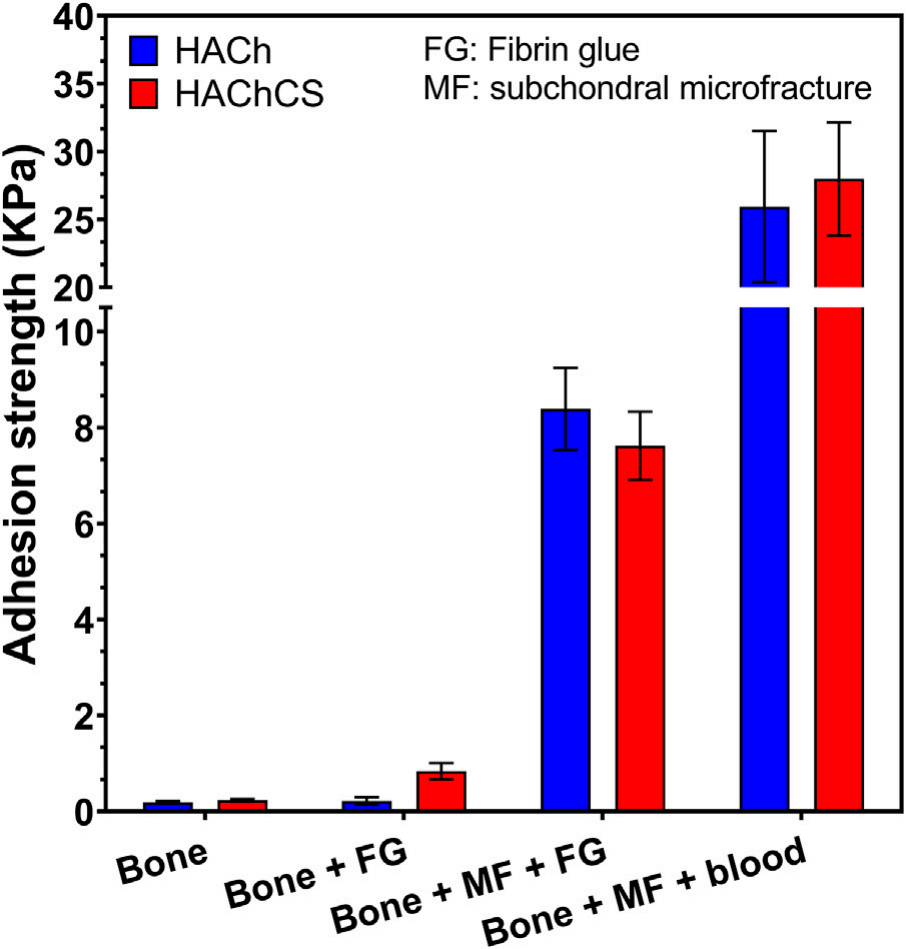
Adhesion strength of the scaffolds to a bone in different conditions. FG: the scaffold was swollen with fibrin glue. MF: A microfracture procedure was applied to the bone. Blood: The scaffold was swollen with blood.

Ch is a natural mucoadhesive and could be a good option to stabilize the scaffold in the microfracture procedure. There are several options to stabilize the scaffold as can it be the use of fibrin glue o surgical adhesives (Frehner and Benthien, 2018; Orth et al., 2020). To measure the mucoadhesive properties of our scaffolds we put in contact the scaffold with bone tissue in different conditions. Figure 4 displays the values of adhesion strength of the scaffolds to bone:

The adhesion of the scaffolds to the bone in absence of microfracture was practically negligible even when fibrin glue was applied. After the application of the microfracture to the bone, the adhesion strength noticeably increased by the augmentation of the contact surface due to the bone holes. In addition, there was also observed an important difference between the use of fibrin glue or blood to seal the scaffold. The adhesion strength was significantly higher in the presence of blood. Moreover, the presence of Ch could accelerate blood coagulations, platelet adhesion and thrombin generation resulting in an increase of the adhesion strength (Hu, 2018). This improvement of the adhesion strength makes this kind of systems good candidates to stabilize the clot coming from the microfracture process in the treatment of cartilage injures.

### 3.4 *In vitro* toxicity and cell adhesion studies

**FIGURE 5:**
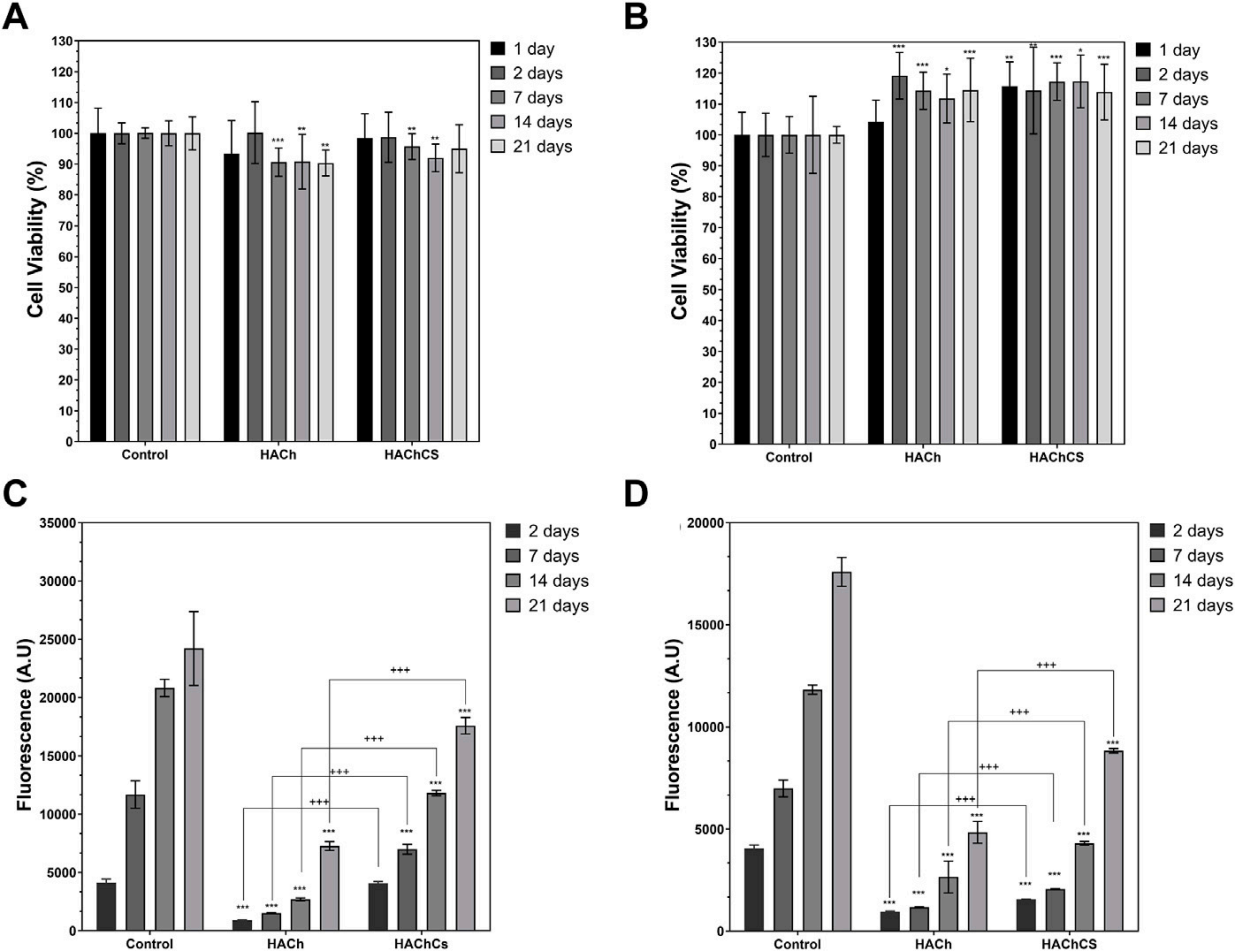
**(A,B)** Cell viability of hACs **(A)** and hOBs **(B)** cultures with HACh and HAChCS scaffolds lixiviates. Significant differences with the control at each time are marked with * (*p < 0.05, **p < 0.01, ***p < 0.001) **(C,D)** Cell proliferation values of hACs **(C)** and hOBs **(D)** seeded on HACh and HAChCS scaffolds. Significant differences with the control (Thermanox^®^ discs) at each time are marked with * (*p < 0.05, **p < 0.01, ***p < 0.001) and between scaffolds are marked with + (^+^ *p* < 0.05, ^++^ *p* < 0.01, ^+++^ *p* < 0.001).

The toxicity of lixiviates coming from the different scaffolds was evaluated on hACs and hOBs (Figures 5A, B). Cell viability of studied samples with hACs (Figure 5A) was not affected on the first 2 days in comparison with the control. After 7 days, significant differences in the viability were observed but in general, the cell viability in presence of any extract was higher than 90%. In the case of hOBs (Figure 5B) cell viability increased in contact with scaffold lixiviates. Ho (2014) observed that the presence of low molecular weight chains of Ch and/or HA in the culture medium improved the cell growth.

Cell adhesion and proliferation on the different scaffolds was evaluated by Alamar Blue assay (Figures 5C, D, Supplementary Figure S2). Cell proliferation increased over time for all the systems but was lower on the scaffolds that on the control. This difference can be ascribed to the surface of the scaffolds, the cells needs more time to adhered on an irregular surface, but after the first 7 days the cell growing on the scaffold surface significantly increase with the time. In SEM pictures (Supplementary Figure S2) we can observe some groups of cells on the surface of the scaffold after 2 days, but after 21 days, the scaffold is completely cover by both tupes of cells. Also in both kinds of cells, the presence of CS on the scaffold improve cell proliferation because CS is one of the main components in ECM and is involved on cell differentiation and proliferation processes (Izumikawa et al., 2014).

### 3.5 Cartilage differentiation on HA based scaffolds

To determine the true potential of our new scaffold in cartilage repair we performed different *in vitro* articular cartilage differentiation assays. The two most common articular cartilage repair interventions (drilling or microfracture surgical procedures), infuse the damaged zone with MSC. We decided to test the differentiation capabilities of our novel biomaterial using a MSC line with and without BMP2 stimulation. This is a reliable way to induce chondrocyte differentiation from MSC lineages (Shea, 2003). We determined the cartilage formation capacity on the three biomaterial combinations (Ch, HACh and HAChCS) and compared to that of a scaffold-free control generating MSC spheroids, a standard protocol for cartilage differentiation (Denker et al., 1995). Then, we measured two aspects of chondrogenesis by quantitative gene expression: 1) predifferentiation towards chondrogenesis measuring Sox9 expression; 2) actual cartilage ECM production measuring aggrecan (Acan) transcription, one of the major components of the cartilage ECM.

**FIGURE 6:**
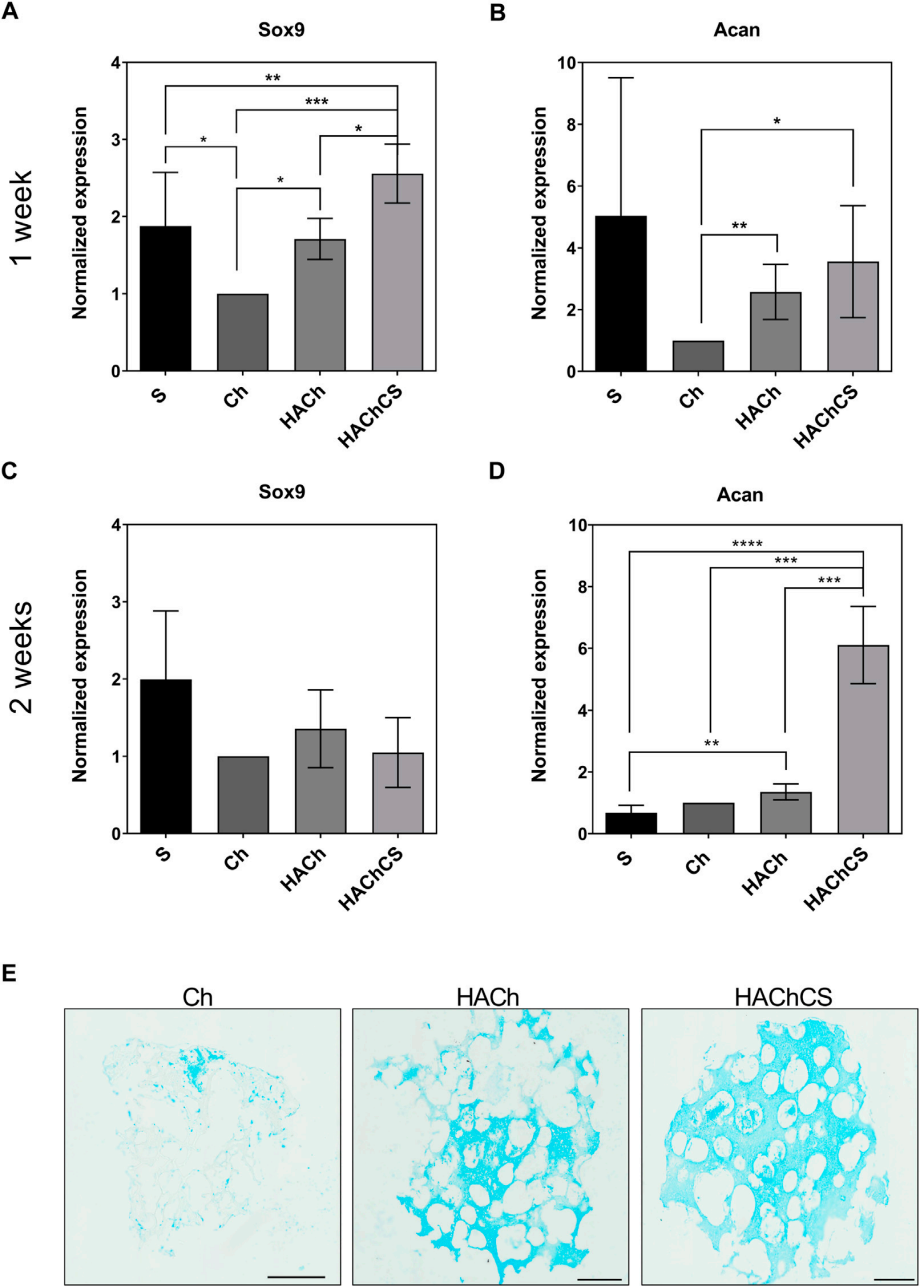
Expression level of Sox9 **(A,C)** and Acan **(B,D)** genes in MSC seeded as spheroids micromass (S) or on scaffolds: Ch, HACh and HAChCS during1 week **(A,B)** or 2 weeks **(C,D)**. Results are expressed as mean ± standard deviation (*p < 0.05, **p < 0.01, ***p < 0.001). **(E)**: Alcian Blue stained sections of Ch, HACh and HAChCS scaffolds seeded with MSC during 1 week. Bars represent 500 µm.

First, we tested the MSC reaction to the different scaffolds to determine if the biomaterials could be pro-chondrogenic activity by themselves without external differentiation factors. Short-term experiments for 1 week of differentiation showed that HAChCS had the best performance giving the highest expression of Sox9, the gene marker for early stages of chondrogenic differentiation, compared even to the standard spheroid method of cartilage differentiation (Figure 6A). Regarding cartilage ECM production, HAChCS also showed higher Acan expression levels than Ch or HACh, with similar performance to standard spheroids (Figure 6B). Mid-term culture of MSC on tested scaffolds during 2 weeks showed similar levels for chondrogenesis marker Sox9 for all biomaterials (Figure 6C). However, at 2 weeks cartilaginous ECM expression significantly increased in HAChCS scaffold (Figure 6D). Histological analysis by Alcian Blue revealed more production of proteoglycans, a major component of cartilage extracellular matrix, and presence of more cells on HAChCS compared to Ch scaffold (Figure 6E). This was confirmed by histological analysis with Alcian Blue, a high affinity staining to cartilaginous proteoglycans, which preferentially binds to cartilaginous ECM (Figure 6E). These results suggest that HA based scaffolds, and more specifically, HAChCS biomaterial inherently induces chondrogenesis in MSC. These results could be due to the release of CS from the scaffolds. CS has shown capacity to activate MSC chondrogenesis and neocartilage deposition (Wang and Yang, 2017).

**FIGURE 7:**
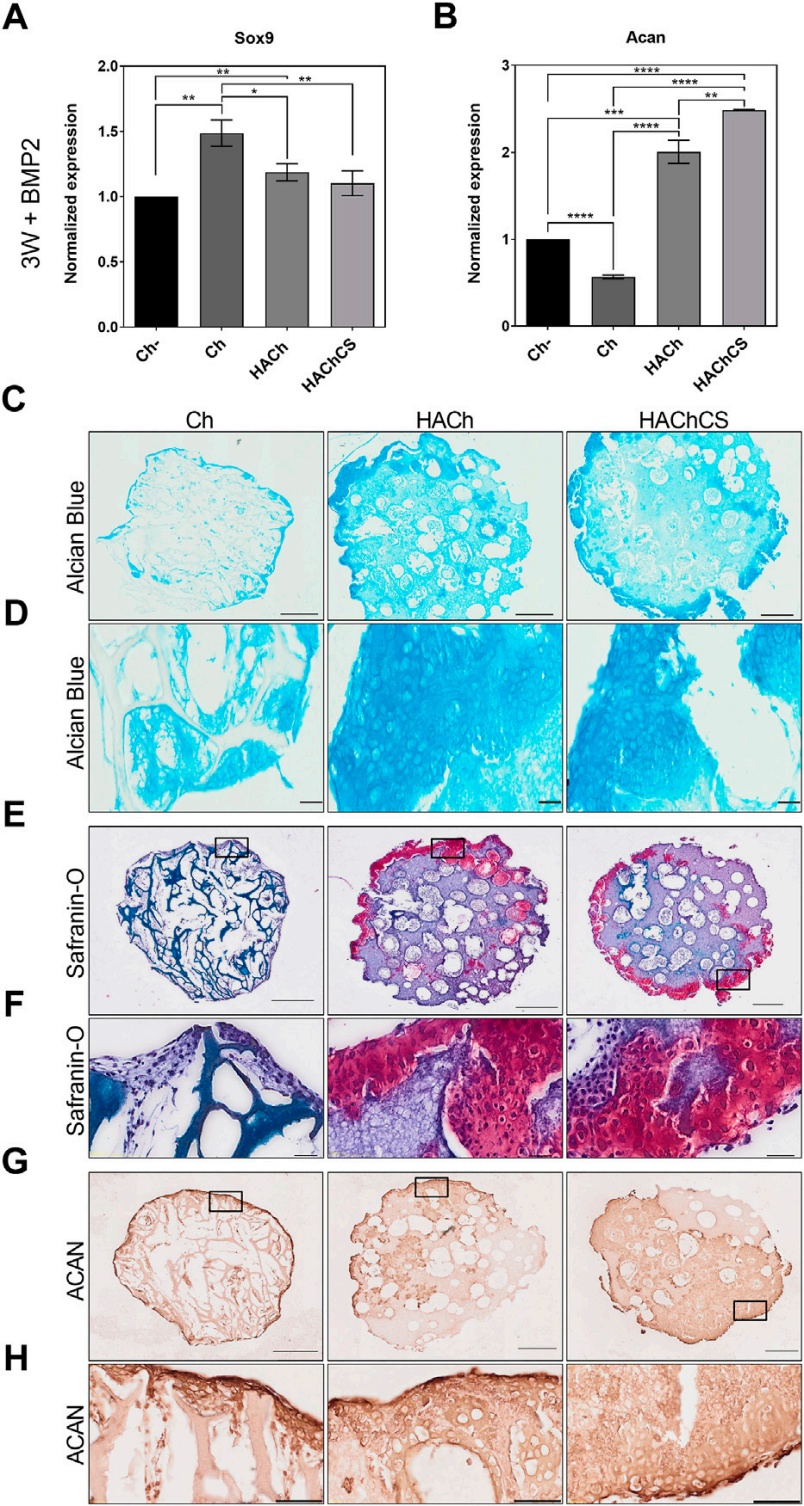
**(A,B)**: Expression level of Sox9 and Acan genes in MSC seeded on Ch without BMP-2 stimulation (Ch-) and Ch, HACh and HAChCS scaffolds after 3 weeks of differentiation with BMP-2 stimulation. Results are expressed as mean ± standard deviation (*p < 0.05, **p < 0.01, ***p < 0.001). **(C)**: Alcian Blue stained sections of Ch, HACh and HAChCS scaffolds with MSC and BMP-2 stimulation for 3 weeks. Bars represent 500 µm **(D)**: High magnification showing well differentiated chondrocytes on the Ch, HACh and HAChCS scaffolds Bars represent 50 µm. **(E)**: Safranin-O stained sections of Ch, HACh and HAChCS scaffolds with MSC and BMP-2 stimulation. Bars represent 500 µm **(F)**: High magnification showing safranin-O staining on the Ch, HACh and HAChCS scaffolds. Bars represent 50 µm. **(G)** ACAN (Aggrecan) immunohistochemical analysis of paraffin-embedded sections of Ch, HACh and HAChCS scaffolds seeded with MSC and BMP-2 stimulation. Bars represent 500 µm **(H)** High magnification showing ACAN (Aggrecan) immunohistochemical analysis on the Ch, HACh and HAChCS scaffolds. Bars represent 50 µm.

Even if scaffolds are prochondrogenic, to produce fully differentiated chondrocytes, cell 3D culture systems need differentiation factors such as BMP-2. To reproduce the microfracture procedure and the articular cartilage environment in the biomaterial, we stimulated MSC with BMP-2 (one of the factors provided by the blood coming from the microfracture of the subchondral bone) allowing cartilage differentiation during 3 weeks (Figure 7). Under these conditions, Sox9 chondrogenic marker showed stabilized levels similar to unstimulated culture after 2 weeks (Figures 6C, 7A), as expected for an early differentiation factor. On the other hand, Acan expression showed higher levels on HA based materials; especially the HAChCS scaffold (Figure 7B. Histological analysis by Alcian Blue (Figures 7C, D) and Safranin-O (Figures 7E, F) showed a production of proteoglycans own of mature articular cartilage (Figure 7C) correlated with well-differentiated chondrocytes with a preferential location on the scaffold surface (Figure 7). Immunostaining of aggrecan, a chondrocyte specific component of cartilaginous extracellular matrix, showed a tissue specific deposition of cells in contact with the HACh and the HAChCS biomaterials, mainly in the surface region of the engineered tissue, confirming its similarities to articular cartilage (Figures 7G, H).

These results suggest that HACh and HAChCS biomaterials promoted *in vitro* chondrogenic differentiation process, as shown by chondrogenic expression markers and cartilage-like tissue formation, and it is expected that they do it in the case of their use in a microfracture procedure.

## 4 Conclusion

Microfracture procedure is a common technique used in cartilage diseases but present several limitations. The use of HAChCS scaffolds on this process could improve the regeneration of articular cartilage. HAChCS presents physico-chemical properties similar to immature cartilage that could allow the regeneration and formation of articular cartilage. Also, the mucoadhesive properties could stabilize the clot coming from the microfracture process and improve the integration with the surrounding tissues. HAChCS did not present toxicity and allowed the adhesion and proliferation of articular cartilage and mesenchymal stem cells. The capacity to release CS activated chondrogenesis process on MSC and the production of proteoglycans without the necessity of any other supplements. Finally, we simulated the environment of a microfracture procedure by addition of BMP-2, one of the common compounds present on the subchondral blood. In these conditions, both HACh and HAChCS scaffolds promoted the chondrogenic differentiation process and cartilage-like tissue formation doing these systems good candidates for their use in the microfracture procedure as well as a scaffold for stem cell and chondro-lineages in next-generation therapies.

## Data Availability

The raw data supporting the conclusion of this article will be made available by the authors, without undue reservation.
