# Supplementary material for: Chemically crosslinked hyaluronic acid-chitosan hydrogel for application on cartilage regeneration
Source: Front Bioeng Biotechnol. 2022 Dec 19;10:1058355. doi: 10.3389/fbioe.2022.1058355 (PMC9806271; doi:10.3389/fbioe.2022.1058355)
Supplement: Supplementary file 1 [file DataSheet1.docx]

Supplementary Material

(A)

(B)

**HACh 2 day**

**HACh 21 day**

**HACh 2 day**

**HAChCS 2 day**

**HAChCS 21 day**

**HACh 21 day**

**HAChCS 2 day**

**HAChCS 21 day**

# Supplementary Tables

**Supplementary Table 1**. EDS analysis on the surface of the different scaffolds.

|  | **Normalized Atomic Composition (wt. %)** | | | | |
| --- | --- | --- | --- | --- | --- |
|  | **C** | **N** | **O** | **S** | **Na** |
| **HACh** | 59.8 ± 6.5 | 23.6 ± 3.1 | 16.6 ± 2.1 | 0 ± 0 | 0 ± 0 |
| **HAChCS** | 48.9 ± 4.6 | 15.5 ± 1.8 | 11.8 ± 1.1 | 0.2 ± 0.2 | 23.6 ± 1.1 |

# Supplementary Figures

**Supplementary** **Figure 1 (A)** Storage (Blue) and loss (Green) moduli evolution from 0.01 to 20 Hz at 25 ºC of HACh (empty symbol) and HAChCS (filled symbol) scaffolds. **(B)** Complex modulus (G*) variation in function of the frequency under a constant load force (5N).


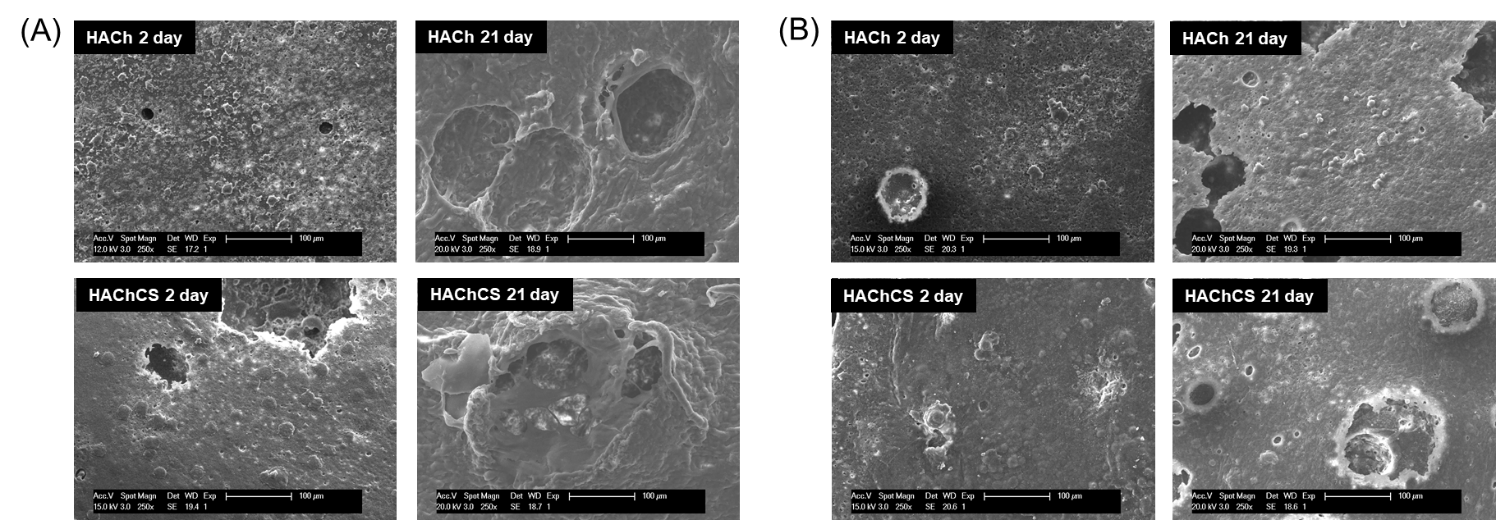


**Supplementary** **Figure 2 (A)** Chondrocyte adhesion on HACh and HAChCS hydrogels after 2 and 21 days. **(B)** Osteoblast adhesion on HACh and HAChCS hydrogels after 2 and 21 days.


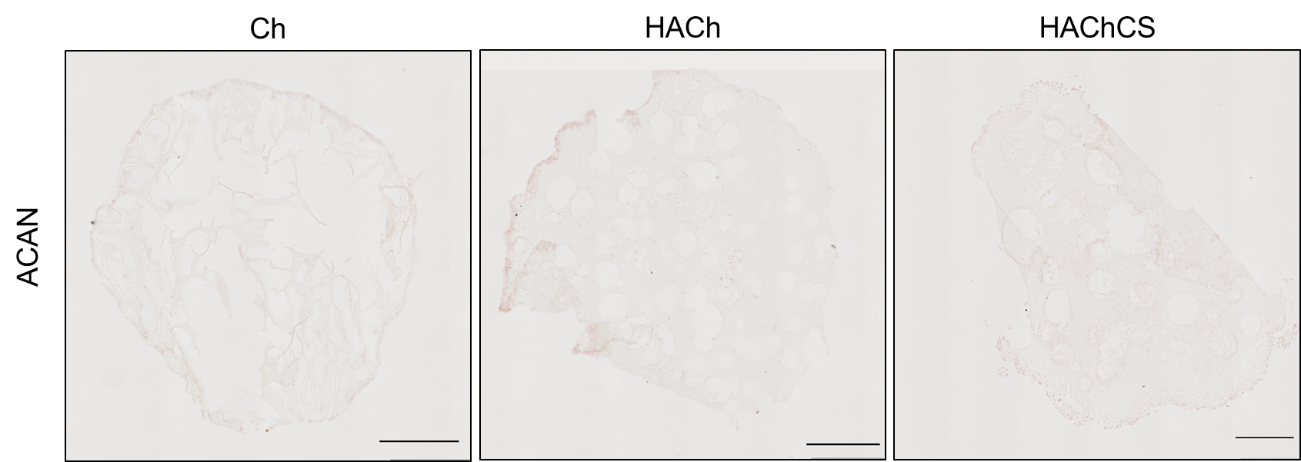


**Supplementary** **Figure 3.** Negative control for immunohistochemical analysis with antigen retrieval and 10 minutes DAB development of paraffin embedded sections of Ch, HACh and HAChCS scaffolds seeded with MSC and BMP-2 stimulation. Bar represent 500 um.
